# “Live” Nanomaterials Process Biomimetic Recognition and Assembly In Vivo

**DOI:** 10.1002/smsc.202300032

**Published:** 2023-10-10

**Authors:** Kuo Zhang, Yuan Li, Bo Huang, Hui Cao, Lei Wang, Hao Wang

**Affiliations:** ^1^ CAS Center for Excellence in Nanoscience CAS Key Laboratory for Biological Effects of Nanomaterials and Nanosafety National Center for Nanoscience and Technology (NCNST) No. 11 Beiyitiao Zhongguancun Beijing 100190 China; ^2^ Department of Materials Physics and Chemistry School of Materials Science and Engineering University of Science and Technology Beijing Beijing 100083 China; ^3^ Department of Orthopedics Xinqiao Hospital Army Medical University Chongqing 400037 China

**Keywords:** nanomaterials, biomimetic, recognition and assembly, in vivo, disease theranostic

## Abstract

The biomimetic strategy has been widely used in multiple fields, and provides huge convenience to human life. For biomedical application in vivo, the importance of biomimetic materials lies on mimicking the natural process/behavior as well as mimicking the structure/ingredient. Herein, recent advances of materials processing biomimetic recognition and assembly behavior for in vivo applications, including the natural source materials and artificial materials, are summarized. The biological processes are divided as recognition and assembly as well as recognition and self‐amplifying assembly. Finally, to conclude, the application of recognition and assembly process‐biomimetic materials in vivo in the future is discussed.

## Graphical abstract

Recent advances of materials processing biomimetic recognition and assembly behavior for in vivo applications, including the natural source materials and artificial materials, have been summarized. The biological processes are divided as recognition and assembly as well as recognition and self‐amplifying assembly. Finally, the article concludes on the application of recognition and assembly process‐biomimetic materials in vivo in the future.

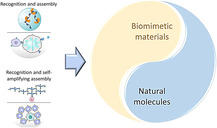

## 1 Introduction

The biomimetic strategy plays an important role in material science, which is widely used in architecture, machine, artificial intelligence, and biomedicine.^[^ ^1^, ^2^, ^3^, ^4^, ^5^, ^6^ ^]^ Biomimetic materials are designed based on structure and function of natural materials, and can be employed as a replacement for natural one. However, the construction of biomimetic materials in vivo remains a huge challenge due to the complex physiological/pathological conditions. Researchers are devoted to designing materials that can mimic natural structures, processes, and functions under specific physiological/pathological conditions to achieve disease therapeutic effect in vivo. Different from ex vivo biomimetic materials, mimicking the process/behavior of nature in vivo is the most important part, which should be paid much attention.

The concept of process‐biomimetic materials, which materials mimic the natural physiological/pathological processes, has been proposed and under extensively studies in the last decade.^[^ ^7^, ^8^, ^9^ ^]^ Recognition and assembly is an important part in process‐biomimetic behaviors. The process of natural recognition and assembly, which leads to multiple physiological/pathological actions, is widely appeared in human body. For example, the recognition and assembly between misfolded protein and molecular chaperone could lead to the repairment of protein, the recognition and assembly between fibronectin and integrin could lead to the fibrillogenesis of fibronectin and subsequent formation of extracellular matrix (ECM), etc.^[^ ^10^, ^11^ ^]^ Therefore, biomimetic materials mimicking natural recognition and assembly have attracted great interests due to their excellent in vivo functions.

In this review, we summarize recent advances of materials mimicking recognition and assembly, and recognition and self‐amplifying assembly behaviors in vivo (**Box** **1**). Not only natural proteins and cells, artificial molecules and self‐assembled structures could be recognized as “live” materials, which perform programmable morphological transformation, directional movement, or other “live” behaviors during the process of recognition and assembly. The natural processes in human body as well as their biomimetic processes are discussed. The composition, behavior, and application of the recognition and assembly biomimetic materials are discussed. Finally, we conclude and prospect the applications of process‐biomimetic materials based on recognition and assembly in vivo in the future.

**Box 1:**
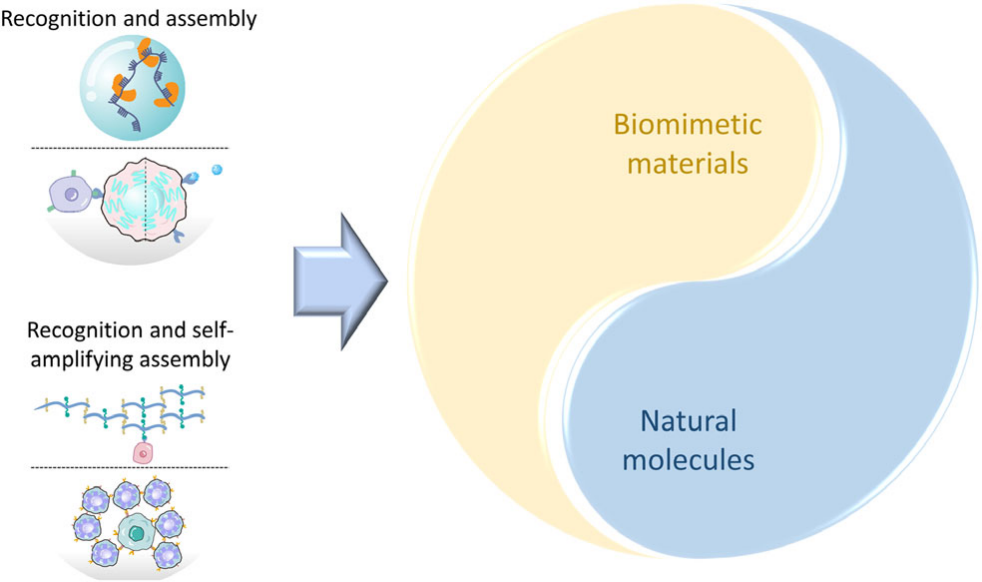
Recognition and assembly materials mimicking processes of natural ones.

## 2 Recognition and Assembly

**Figure 1:**
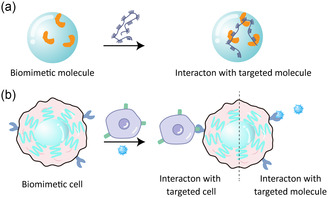
Recognition and assembly. a) Biomimetic molecules (e.g., molecular chaperones) or b) cells (e.g., RBCs, APCs, and WBCs) work via recognizing and assembling with targeted molecules or cells.

The recognition and assembly of biomolecules could lead to inhibition of the misfold of protein,^[^ ^11^ ^]^ absorbing of toxin by red blood cells (RBCs),^[^ ^12^ ^]^ immune escape of RBCs,^[^ ^13^ ^]^ activation of T‐Cell,^[^ ^14^ ^]^ localization of white blood cells (WBCs) in inflammatory position,^[^ ^15^ ^]^ etc. Inspired by natural process of recognition and assembly, a series of biomimetic materials are prepared for similar functions to natural ones (**Figure** **1**).

### 2.1 Molecular Chaperones Repair Natural Proteins

Molecular chaperones are critical components in cellular quality control system, which play an important role in controlling unnecessary misfolding of protein and maintaining the stability of complex protein structures.^[^ ^11^, ^16^ ^]^ Proteins, such as human chaperone Hsp70, apolipoprotein E, and Spy, could bind and form stable complex with exposed hydrophobic residues on abnormal proteins (Figure 1a).^[^ ^17^, ^18^ ^]^ It is well known that molecular chaperone Spy could bind protein Im7 to induce the fold of Im7. Spy interacts with unfolded Im7 (Im7_U_) to form a compacted structure with two equivalent interaction surfaces, inducing the fold of Im7_U_. The interaction between Spy and Im7 is weak, subsequently leading to the disassembly.^[^ ^19^ ^]^ These evidences suggest molecular chaperones are promising therapeutic agents for the treatment of neurodegenerative diseases, such as Alzheimer's disease (AD), Parkinson's disease, etc.^[^ ^11^, ^20^, ^21^, ^22^ ^]^

The principle of molecular chaperone is to modulate the assembly of natural proteins with a balance of hydrophobic and hydrophilic interactions. Biomimetic molecular chaperone may exhibit inhibition of the assembly of proteins for disease therapy with similar structures to natural molecular chaperone.^[^ ^23^ ^]^ For example, polymeric structures such as poly(β‐amino ester)‐*block*‐poly(ε‐caprolactone) (PAE‐*b*‐PCL) and poly(ethylene oxide)‐*block*‐poly(ε‐caprolactone) were utilized for inhibition of amyloid‐β^[^ ^24^ ^]^ and insulin^[^ ^25^ ^]^ through recognition and assembly, respectively. In addition, benzimidazole functionalized polyfluorene (PFBZ) was confirmed to bind and assemble with amyloid‐β with unprecedented selectivity for inhibiting the interaction between amyloid‐β and cells.^[^ ^26^ ^]^ The biomimetic molecular chaperone could be designed with natural recognizing sequence such as Lys–Leu–Val–Phe–Phe (KLVFF) for AD treatment.^[^ ^27^, ^28^ ^]^

Natural molecular chaperone aimed to inhibit abnormal assemble of proteins, especially amyloid‐β. Similarly, the biomimetic molecular chaperone could recognize and assemble with aggregatable proteins. The biomimetic molecular chaperone should have proper binding as well as dissociation ability.^[^ ^29^ ^]^ Supramolecular biomimetic molecular chaperones may be promising for disease therapy due to their large number of binding sites. Meantime, the biosafety of the biomimetic chaperone based on synthesized polymer should also be taken into consideration.

### 2.2 RBCs Capture Toxin

RBCs are a class of nutrition transporting cells in blood, which are also excellent binding site for toxins. Pore forming toxins (PFTs) are toxic structures, which is secreted by bacteria and could target host cells for further damage (Figure 1b).^[^ ^30^ ^]^ Two kinds of assembly mechanism between toxin and membrane of RBCs are purposed.^[^ ^31^ ^]^ The hydrophobic β‐tongue in toxin binds and inserts into membrane of RBCs, followed by the formation of assembled complex with helix structure. The assembled complex further undergoes a downward movement to form a pore structure, which is named as “pre‐pore” model. Another pore formation model is “growing‐pore” model, in which toxin exhibits a conformational transformation after binding membrane of RBCs through β‐tongue. The unstable complex further forms a pore through oligomerization. In a word, the assembly between toxin and cell membrane forms a pore on the cell membrane, leading to the death of RBCs.

Toxin could lead to the apoptosis of RBCs. Therefore, the artificial RBCs were developed to replace natural RBCs from being destroyed. Backbone, such as polylactic acid–glycolic acid (PLGA), was utilized to maintain the shape of artificial RBCs, which were utilized for broad‐spectrum antivirulence and related areas (Figure 1b).^[^ ^32^, ^33^, ^34^ ^]^ Besides, functional structures, such as acetylcholinesterase and α‐hemolysin (Hla), were successfully loaded on the membrane, performing detoxification of organophosphate and protective immunity to *Staphylococcus aureus*, respectively.^[^ ^35^, ^36^, ^37^ ^]^ The Hla could be encapsulated into membranes of RBCs and functioned as nanovaccine.^[^ ^38^, ^39^ ^]^

The natural RBCs could deliver oxygen to cells in need. However, they would be easily destroyed by toxins, which may lead to damage to human body. Artificial RBCs were composed of membrane of natural RBCs and artificial structures as backbones. Therefore, the artificial RBCs were not able to carry oxygen. In the meantime, they could act as target of toxin to prevent natural RBCs from being attacked. The artificial RBCs could further load therapeutic drugs, leading to enhanced therapy effect. In addition, artificial RBCs may preabsorb toxin as nanovaccine against bacterial infection.

The binding affinity between artificial RBCs and different toxin should be carefully evaluated. The artificial RBCs were expected to perform higher binding affinity to toxin comparing with natural RBCs in order to absorb as much toxin as possible. The in vivo distribution as well as degradation and metabolism of artificial RBCs remained unsolved.^[^ ^40^ ^]^ The backbone in the artificial RBCs is important, which should maintain the stability during the recognition and assembly between toxin and cell membrane.

### 2.3 RBCs Exhibit Long‐Term Circulation

RBCs exhibit long‐term circulation, which is due to the expression of CD47 on the surface of RBCs membrane.^[^ ^41^ ^]^ The CD47 assembles with the extracellular IgV domain of signal regulatory proteins α (SIRPα) on macrophage, leading to the release of “don't eat me” signal and resultant long‐term circulation of these cells (Figure 1b).^[^ ^13^, ^42^ ^]^ It is confirmed that the residue Cys‐15 binds Cys‐241 or Cys‐245 to form disulfide bond, which leads to the conformational transformation of CD47 and subsequent increase of binding affinity to SIRPα.^[^ ^43^ ^]^ Therefore, the membranes of RBCs could be utilized as carriers to load cargoes for a highly efficient delivery with prolonged circulation time in blood.^[^ ^44^, ^45^ ^]^

A large amount of biomimetic RBCs have been developed for encapsulating cargoes. Structures such as mesoporous silica,^[^ ^46^ ^]^ chitosan nanogels,^[^ ^47^ ^]^ PLGA,^[^ ^48^ ^]^ etc. could act as backbone for loading therapeutic agents.^[^ ^49^, ^50^ ^]^ Chemotherapy, photodynamic therapy, and photothermal therapy of tumor may be achieved through the encapsulation of doxorubicin (DOX),^[^ ^51^ ^]^ indocyanine green,^[^ ^52^ ^]^ and melanin,^[^ ^53^ ^]^ respectively. Recently, novel nanoscale structures, such as metal–organic frameworks (MOFs) were introduced into the membrane of RBCs for tumor treatment through microwave dynamic therapy.^[^ ^54^ ^]^ The drug‐loaded biomimetic RBCs exhibited immune escape ability, leading to long‐term circulation and subsequent improved effect on tumor therapy.

The therapeutic agents could be easily uptaken by reticuloendothelial system (RES) and cleared by immune system,^[^ ^55^ ^]^ leading to short‐term circulation. Membranes of natural RBCs were utilized to encapsulate therapeutic agents for immune escape with prolonged circulation time, contributing to improved therapeutic effect. The biosafety of artificial RBCs should be carefully evaluated to avoid unneeded accumulation in normal tissues. It was found that porous structures were frequently used as backbone on forming biomimetic RBCs for loading more therapeutic agents. Further studies may be focused on increasing loading amount and decreasing leakage amount of theranostic agents, as well as avoiding infections and blood type incompatibility.

### 2.4 Antigen‐Presenting Cells Activate T‐Cells

The activation of T‐cells by antigen‐presenting cells (APCs) is achieved through the recognition and assembly between the two cells. The activation process consists of two steps. The foreign peptide binds to major histocompatibility complex (MHC) on the surface of APCs to form a peptide–MHC complex, which further binds to T‐cell for activating T‐cells. Besides, CD80 and CD86 on the membrane of APCs could also assemble with CD28 on the surface of T‐cells for the activation of T‐cells.^[^ ^56^ ^]^ The key amino acids during the assembly behavior between CD80 or CD86 and CD28 are identified through molecular dynamic simulations.^[^ ^57^ ^]^

For the purpose of activation of T‐cells, artificial APCs (aAPCs), which are originated from the encapsulation of goods into membrane of natural APCs, should accumulate in lymph nodes and spleen with large amount of T‐cells. The size of aAPCs was crucial, in which a diameter of 100–200 nm was most suitable to avoid uptake by macrophage or liver in circulation.^[^ ^58^, ^59^ ^]^ The shape, especially the local curvature, was critical for the binding between aAPCs and T‐cells and reducing unneeded internalization, in which ellipsoid morphology exhibited higher binding affinity and decreased internalization comparing with sphere or filamentous worm morphology.^[^ ^60^, ^61^ ^]^ Large amount of aAPCs were designed and utilized for disease therapy.^[^ ^62^ ^]^ For instance, anti‐PD‐1 monoclonal antibody‐coated and PLGA‐encapsulated aAPCs were utilized for immunotherapy of cancer.^[^ ^63^, ^64^ ^]^ Immune‐related factors, such as CD80, CD58, CD54, etc., could also be expressed on membrane of aAPCs for improved immunotherapy.^[^ ^65^ ^]^

It should be noted that the size and shape of aAPCs could affect the therapeutic effect. The surface of aAPCs should be precisely modified for precise applications. Besides, the synthesis technique should be updated to decrease the participation of organic solvent for improved biosafety and high product purity. In addition, undesired immune response should be carefully monitored.

### 2.5 WBCs Localize Inflammatory Position

Many harmful substances, such as bacteria, virus, and chemicals, as well as injury may cause inflammation in human body.^[^ ^66^, ^67^ ^]^ Immune system could recognize the harmful substances as antigen to initialize inflammatory response.^[^ ^68^, ^69^ ^]^ Cell adhesion molecules as well as chemotactic proteins are essential for the recruitment of WBCs to inflammatory area.^[^ ^70^ ^]^ Binding between selectin glycoprotein on endothelial cell and ligands of selectin on WBCs contributes to the rolling of WBCs along vessel wall.^[^ ^71^ ^]^ Subsequently, the recognition and assembly between integrin LFA‐1 on T cells and intercellular adhesion molecule 1 (ICAM‐1) on WBCs could lead to the localization of WBCs to inflammatory position (Figure 1b).^[^ ^72^ ^]^ More specifically, the *α*_L_ I domain in integrin recognizes and assembles with the first domain (D1) in ICAM‐1.^[^ ^73^ ^]^ The recognition and assembly is able to effectively eliminate inflammatory.^[^ ^15^, ^74^, ^75^ ^]^

The membrane of natural WBCs was utilized to encapsulate cargoes for the treatment of inflammatory precisely (Figure 1b). For example, Resolvin D2 (RvD2) was loaded into membrane of neutrophil to form nanovesicles with diameter of about 200 nm for alleviating inflammation and protecting brain after ischemia/reperfusion injury.^[^ ^76^ ^]^ In another case, superparamagnetic iron oxide nanoparticles (SPIO) were encapsulated into membrane of WBCs, which could locate ICAM‐1 overexpressed tumor cells for assembly and subsequent visualization of inflammation area as well as alleviating inflammation in vivo.^[^ ^77^, ^78^ ^]^ It can be concluded that the membrane of WBCs could be utilized as carriers for therapeutic agent with multiple functions. Studies should be concentrated on the precise modulation in size of artificial WBCs to adapt different pathological environments.

The recognition and assembly behavior is widely observed in human body, which participates in multiple biological processes. Inspired by the natural process, artificial materials, especially artificial cells, are developed.

## 3 Recognition and Self‐Amplifying Assembly

**Figure 2:**
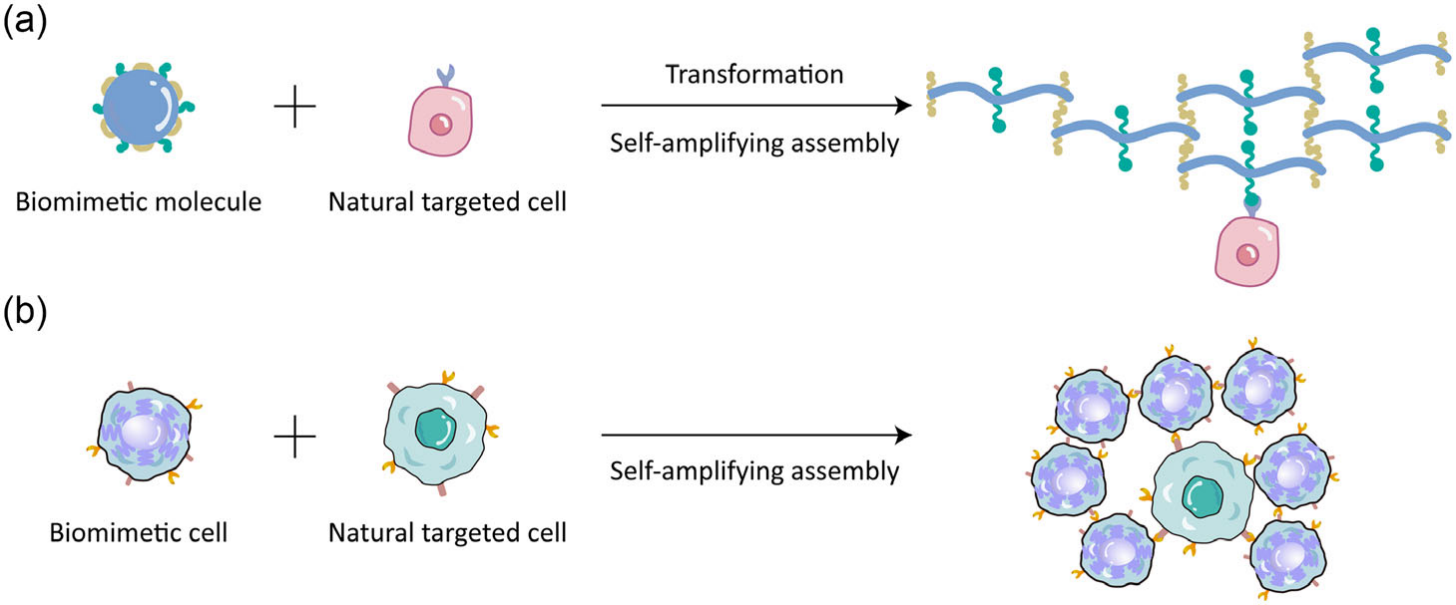
Recognition and self‐amplifying assembly. a) Biomimetic molecules (e.g., HD‐6, fibronectin, and laminin) or b) biomimetic cell (e.g., platelets and tumor cells). The recognition and assembly between ligand and receptor results in the morphological transformation and exposure of new self‐recognition and assembly sites, leading to the recognition and self‐amplifying assembly.

There is a kind of recognition and assembly, named as recognition and self‐amplifying assembly, the process of which is widely observed in human body. In the process of self‐amplifying assembly, the as‐formed assembly structures could expose more (new) binding sites for interacting with more self‐assembly ones (**Figure** **2**).^[^ ^79^, ^80^, ^81^, ^82^ ^]^ In this section, the recognition and self‐amplifying assembly behaviors, including the formation of human defensin‐6 (HD‐6) nanofibers (NFs), fibrillogenesis in ECM, the aggregation of platelets, and tumor cells, are introduced. The corresponding mimicking materials are discussed with their designs and applications.

### 3.1 HD‐6 Captures Bacteria

HD‐6 is an antimicrobial peptide, which is expressed and secreted by Paneth cells.^[^ ^79^ ^]^ The HD‐6 could recognize invaded microbes and inhibit the infection.^[^ ^83^ ^]^ The proposed mechanism is that HD‐6 could self‐assemble into fibrous networks and further capture microbial pathogens, which contributes to the blockage of bacteria invasion.^[^ ^84^ ^]^ More specifically, four monomers of HD‐6 could form a fibrous structure through hydrophobic pocket of HD‐6, in which Phe2, Phe29, and Leu32 in two monomers and Val22, Met23, and Ile25 in the other two monomers contribute to the forming of fibrous structure. Besides the recognition and assembly of HD‐6, the formed HD‐6 fibers with exposure of hydrophobic residues further induce self‐assembly of HD‐6, i.e., self‐amplifying assembly. Contrary to normal human defensins, HD‐6 preferred to trap microbes rather than attack them.

Inspired by the process of natural HD‐6 capturing bacteria, a HD‐6 mimic peptide (HDMP) was developed. The HDMP exhibited antibacteria effect in a similar manner, in which nanoparticulate HDMP could bind lipoteichoic acid on gram‐positive bacteria and assembled into nanofibrous structures in a biomimetic self‐amplifying mode. The bright green fluorescence signal of HDMP NFs could contribute to diagnostics, and subsequently inhibiting the invasion of bacteria (Figure 2a).^[^ ^85^ ^]^

The smart design of HDMP is a promising strategy for antibacteria. The targeting sequence could be replaced by other sequences, which may be utilized for treating different types of invasion microbes, such as gram‐negative bacteria, fungi, etc. Besides, the direct killing ability of bacteria might be developed through the addition of direct killing ligand.

### 3.2 Fibrillogenesis of Fibronectin Forms ECM

Natural ECM consists of proteoglycans and proteins with fibrous structure, such as fibronectin (FN) and laminin (LN).^[^ ^86^ ^]^ The proteins are secreted by cells and could self‐assemble into uniformed fibrous networks (Figure 2a). Naturally, FN binds to integrin and initiates the fibrillogenesis. Besides, the integrin clusters and FN further expose FN binding sites, promoting FN–FN interactions and fibrillogenesis, i.e., the soluble dimmer of FN transforms into fibrous structure through binding‐induced fibrillogenesis (BIF).^[^ ^87^ ^]^ Similarly, LN could also form fibrous structure through BIF.^[^ ^88^ ^]^ In addition, the acidic microenvironment is confirmed to accelerate the fibrillogenesis of LN through inducing the protonation of histidine residues and subsequently changing the hydrophilic–hydrophobic balance.^[^ ^89^ ^]^ In addition, the mechanical forces from actin filament inside cells through protein–ligand and receptor–protein on the surface of cell membrane participates in the self‐assembly process.^[^ ^80^ ^]^

Inspired by the fibrillogenesis of natural FN or LN through BIF behavior, the FN/LN‐mimicking peptides were designed, which could bind integrin α_V_β_3_ (Ca^2+^) in solution or integrin α_V_β_3_ in vivo to form nanofibrous structure.^[^ ^90^, ^91^ ^]^ The preformed NFs further provided more FN/LN‐mimicking peptide binding sites for self‐amplifying self‐assembly, leading to the formation of fibrous network.^[^ ^92^ ^]^ The biomimetic FN/LN recognized tumor with overexpressed α_V_β_3_ and formed artificial ECM, leading to the migration and invasion inhibition of tumor cells. More importantly, the NFs exhibited long‐term retention and inhibition effect due to the ordered and stable β‐sheet structures.^[^ ^93^, ^94^, ^95^ ^]^

Natural FN/LN could form fibrous structure in ECM, leading to the mobilization of cells and inhibiting cell migration with relative poor enzyme degradation resistance. Biomimetic FN/LN peptides could bind specific receptors and form stable NFs through BIF, showing better enzymatic stability.^[^ ^96^, ^97^ ^]^ Biomimetic FN/LN peptides may perform natural functions, such as migration inhibition of endothelial cells, as well as other functions, such as embolization of blood vessel, suggesting wide applications of biomimetic ECM. The BIF behavior of FN/LN biomimetic peptides could be achieved through binding many other receptors on cell membranes, which may result in therapeutic agents for various diseases. Further studies may focus on the finding of new targets and new peptide ligands for diverse disease therapeutics.

### 3.3 Platelets Aggregate and form Thrombus

Platelet is an important component in blood, which shows multiple functions. Platelets could exhibit long circulation time in blood and could perform immune escape ability through reducing the clearance by reticuloendothelial system with the help of expressed CD47 on the membrane.^[^ ^98^ ^]^ More importantly, platelet could aggregate and form thrombi through recognition and self‐amplifying assembly.^[^ ^81^ ^]^ The process of aggregation and forming thrombi of platelet is as follows.^[^ ^99^ ^]^ First, platelet target and bind to collagen, which is located on the damaged endothelial cells and transforms into satellite‐like structure through ligand–receptor interaction. The morphology transformation process enables transformed platelet exposing more binding site, which could attract more platelet and induce the self‐amplifying assembly of platelet. The soluble fibrinogen was also transformed into insoluble fibrin with fibrous morphology in the meantime. Finally, the platelet aggregate, insoluble fibrin, and RBCs tangle together and form thrombus.

A series of nanostructures mimicking the recognition and self‐amplifying assembly of natural platelet were prepared. Inorganic SPIO were conjugated with plasma protein targeting peptide CREKA, which could target and bind with vessel walls and tumor stromal for inducing clotting in vivo and subsequent self‐amplifying assembly by emerging more binding sites for tumor imaging (Figure 2b).^[^ ^100^ ^]^ Besides, peptidic platelet‐like nanoparticles (pNPs) were developed for tumor embolization. The pNPs bind endothelial cells to form NFs through BIF, exposing more pNPs binding sites for self‐amplifying assembly, leading to the embolization of blood vessel of tumor through capturing RBCs.^[^ ^96^ ^]^ Similarly, peptide sequence could be modulated to perform hemostasis and wound healing properties.^[^ ^101^ ^]^

It could be observed that the peptide participated in different types of biomimetic process, which may be due to the excellent binding affinity and biocompatibility. The peptide mimicking natural clotting may concentrate on the self‐targeting and self‐assembly.

### 3.4 Tumor Cells Accumulate and Aggregate

The homotargeting ability of tumor cells has been under extensive investigation.^[^ ^102^ ^]^ Researchers found that the interaction between T antigen core 1 disaccharide and the galectin‐3 is the basic signal pathway for the recognition between tumor cells.^[^ ^82^ ^]^ Endothelium‐expressed galectin‐3 could bind to the T antigen on the tumor cells, which lead to aggregation of tumor cells on the endothelium of blood vessels. Furthermore, galectin‐3 translocates to tumor cells and triggers aggregation of tumor cells. The reasons of galectin‐3 nuclear translocation are unknown. Surface adhesion molecules, such as N‐cadherin and epithelial cell adhesion molecules, are also responsible for the homologous adhesion behavior of tumor cells in similar manner.^[^ ^103^ ^]^ The targeting and binding among tumor cells also exhibit a self‐amplifying aggregation process, which is similar to the aggregation of platelet.

Multiple artificial cells were successfully utilized form tumor imaging and therapy through homologous targeting. The selection of membrane of tumor cells was focused on cells with high migration and invasion ability, such as B16‐F10, MDA‐MB‐435, HCT 116 cell lines, etc. (Figure 2b).^[^ ^104^, ^105^ ^]^ The backbone was also important for the stabilization of artificial cells. Therefore, traditional structure such as PLGA and novel structure such as MOFs and upconversion nanoparticles were encapsulated into membrane of tumor cells.^[^ ^105^, ^106^ ^]^ In addition, cargoes, including indocyanine green (ICG), catalase (CAT), etc., could further be loaded into the backbone for improving therapeutic efficacy, such as photothermal therapy and inducing cell apoptosis, respectively.^[^ ^103^, ^106^ ^]^ Artificial cells could not only perform homologous targeting ability, but also immune escape ability, which ultimately led to precise tumor therapy.^[^ ^107^ ^]^

Similar to other artificial cells, the principal motifs in artificial cells are cell membrane, backbone, and therapeutic agents. As confirmed, the membrane should express specific receptors for homologous targeting. The backbone should support and stabilize the structure of artificial cells. Meanwhile, porous structure is highly recommended, which contribute to loading more cargoes for tumor imaging or therapy.

Inspired by nature, a series of biomimetic structures are developed, forming aggregates or fibrous structures in situ. Large assemblies exhibit improved therapeutic effects comparing with the nonaggregatable controls with long‐term retention in disease site. The assembly behavior, especially self‐amplifying assembly, should be precisely modulated carefully to prevent the nonspecific damage of normal organism. Most natural “materials” are secreted directly in the localized disease area. However, artificial “live” materials are commonly administrated through i.v. injection, which should be optimized for high‐efficient transportation in vivo.

## 4 Conclusion and Prospects

We have summarized recent advances of process‐biomimetic nanomaterials on the recognition and assembly in vivo. “Live” materials refer to different aspects, such as peptides, cell membranes, and artificial polymers, which could mimic natural structures, processes, and functions. Many issues could affect the properties and bioeffects of process‐biomimetic materials, including the complexity of manufacturing, biocompatibility, biosafety, long circulation ability, targeting ability, etc. For the purpose of in vivo applications, one should not only consider the theranostic effect of the biomimetic materials, but also ensure the biosafety of the biomimetic materials. Precise targeting ability is highly recommended to avoid unnecessary accumulation and cytotoxicity in normal tissues. Assembly behavior should be precisely triggered by stimuli, such as pH, receptors, etc., at specific site and time, leading to improved biosafety. Based on these considerations, process‐biomimetic materials should exhibit following characteristics: specificity to target site, precise recognition and assembly in vivo, and minimized systematic toxicity.

Researchers have made great efforts to construct functional biomimetic nanostructures. On the one hand, the membrane of natural cells is utilized to encapsulate cargoes with/without surface modification to obtain artificial cells. The artificial cells could maintain the recognition and assembly ability of natural cells with multiple functions such as biocompatibility, biosafety, long circulation ability, immune escape ability, and homotargeting ability. The biomimetic cells could perform precise theranostic ability in vivo. On the other hand, biomimetic materials based on artificial polymers, peptides, etc., mimicking the natural recognition, and assembly behavior are developed. For example, mimicking recognition and self‐amplifying assembly of natural HD‐6, HD‐6 mimicking peptide could capture gram‐positive bacteria. Platelet mimic peptide could form clots for vessel embolization through mimicking coagulation of platelet. The high performance over natural ones is expected with modulation of composition, size, and morphology of these biomimetic materials.

In the present stage, researchers are devoting to the investigation of biomimetic materials mimicking natural recognition and assembly for improved therapeutic effect in vivo. However, few explorations have been employed for clinical applications. Further effort should work on the clinical trials for biomimetic materials. To achieve this target, many issues need to be improved, i.e., the establishment of the standard of preparation technique and evaluation system. Meanwhile, performance and degradation of biomimetic materials in vivo should be monitored. Normally, natural proteins, lipids, peptides, etc. could be degraded by various enzymes easily. Biodegradable polymers, which could contribute to the improvement of biosafety, are highly recommended to serve as artificial process‐biomimetic materials. However, it should be noted that the degradation rate of process‐biomimetic materials needs to be precisely modulated in specific region. It is known that fast degradation of process‐biomimetic materials may lose therapeutic efficacy. More importantly, the mechanism of natural recognition and assembly of these artificial biomimetic materials should be carefully studied. These efforts could contribute to increasing the possibility of biomimetic materials into clinical trials. We believe the human health could benefit from these biomimetic materials.

## Conflict of Interest

The authors declare no conflict of interest.
